# OA10 Post-partum haemophagocytic lymphohistiocytosis

**DOI:** 10.1093/rap/rkae117.010

**Published:** 2024-11-05

**Authors:** Bethan Goulden, Ian Giles, Kate Wiles, Alexis Jones

**Affiliations:** University College London, London, United Kingdom; University College London, London, United Kingdom; Royal London Hospital, London, United Kingdom; University College London Hospital, London, United Kingdom

## Abstract

**Introduction:**

Haemophagocytic lymphohistiocytosis (HLH) is a hyperinflammatory syndrome which can progress to multi-organ dysfunction and death. Triggers include rheumatological disease, malignancy, and infection. Successful management requires early recognition, immunosuppression, and treatment of the underlying driver.

Case reports suggest pregnancy may trigger HLH although the mechanism is uncertain. Between 2013-2021, 8 mothers died in pregnancy and the post-partum period in the United Kingdom (UK) secondary to HLH. This suggests that it accounts for around 1% of maternal deaths but the numbers who survive remain unaccounted for and thus its overall incidence and mortality risk remains uncertain.

**Case description:**

A 41-year-old female presented with persistent fever 10 days after a Caesarean delivery of her second child at term. She had a small, resolving area of cellulitis area near to the surgical incision. Cultures of urine, blood, wound, and vaginal swabs were unremarkable, and imaging revealed no infective source. Despite more than 10 days of broad-spectrum antibiotics, she remained febrile. On day 13 post-partum she was afebrile for 24-hours and was discharged home with a further 4-days of oral antibiotics. Ferritin was normal.

She re-presented 20 days post-partum unwell with fever. Examination, microbiological tests, transvaginal ultrasound, and echocardiogram did not reveal a source of infection. Chest x-ray demonstrated bilateral infiltrates and blood tests a raised CRP and mild anaemia with normal white cell and platelet count. Broad spectrum antibiotics were re-commenced, alongside anti-viral therapy for possible herpes simplex virus (HSV). Despite this treatment she rapidly deteriorated and 24 hours later was persistently febrile, hypotensive, increasingly hypoxic, coagulopathic and pancytopaenic. Ferritin was now >2000μg/L and rising. On day 23 post-partum she was intubated requiring 100% FiO2 and assessed in preparation for extra-corporeal membrane oxygenation. The question was then raised: could this be HLH?

Haematology review felt HLH to be unlikely based on initial H-score but rheumatologists, intensivists and obstetricians sought advice from an obstetric physician in the local Maternal Medicine Network (MMN). The obstetric physician obtained urgent input from an HLH team and methylprednisolone and anakinra were commenced the same day. Within 6 hours she was afebrile, vasopressors were weaned, oxygen requirements reduced and within 4 days she was extubated.

She was transferred to a tertiary centre for further investigation but no infective, malignant, autoimmune, or genetic cause of HLH was identified. She was discharged home on day 36 post-partum.

**Discussion:**

HLH must be considered in people with fever, falling cell counts and hyperferritinaemia. Even if deemed unlikely following an initial low H-score, ongoing vigilance is advised and immunosuppression may need to begin prior to confirmation of diagnosis. This requires awareness among all teams who may encounter these patients, and expert, multidisciplinary decision-making.

Due to concern about sepsis, there was disagreement about immunosuppressing the woman described in this case. Some clinicians felt further investigations (e.g., bone marrow biopsy) were required, others feared she was too unwell to delay. They appropriately sought further advice from the tertiary centre for HLH. This intervention was lifesaving. Whilst bone marrow biopsy aids in confirmation of HLH and identification of specific drivers (e.g., lymphoma) – it is just one part of the diagnostic assessment and is not a pre-requisite for treatment. The skills of a Rheumatologist in inflammation, immunosuppression, and navigating diagnostic uncertainty such as this make them a vital member of any HLH team.

No infective, malignant, or genetic cause of HLH was identified in this woman. She was positive for anti-Ro antibodies, but no other autoimmune features were identified. A pragmatic management regime was adopted with steroid and anakinra wean as an outpatient, and by one-year post-partum all immunosuppression was discontinued. On further questioning she reported a similar albeit much milder experience following the birth of her first child: recurrent fevers and a label of post-partum sepsis for which no source was identified. We propose that whilst a possible initial infection (e.g., cellulitis) and degree of immune perturbation (i.e., asymptomatic anti-Ro antibodies) were identified, the immunological shifts occurring post-partum are likely to have been contributory to this case of pregnancy-associated HLH.

We would welcome feedback from other teams on their experience of caring for pregnancy-associated HLH – particularly strategies for addressing barriers for recognition and management?

**Key learning points:**

• HLH should be considered in the presence of the three F’s – Fever, Falling cell counts and hyperferritinaemia

• It is not enough to *think sepsis* - clinical teams must think *beyond bacterial* sepsis in people who are critically unwell, febrile, and not responding to antibiotic therapy or when no bacterial source is identified.

• Differential diagnoses in the setting of pregnancy include non-bacterial sepsis (e.g., disseminated HSV), HLH, or other inflammatory syndromes with a predilection for pregnancy and the post-partum period (e.g., catastrophic anti-phospholipid syndrome, thrombotic thrombocytopaenic purpura)

• Critically unwell pregnant and post-partum females require multi-disciplinary expertise, including from:

• Local MDTs of obstetricians, obstetric anaesthetists, midwives, obstetric pharmacists, intensivists, obstetric physicians, and key specialty teams (e.g., rheumatologists, haematologists)

• Additional support from the wider MMN (available throughout England) and in the case of HLH, from the national HLH MDT (based in London and Sheffield)

• There remain many unknowns for pregnancy-associated HLH – the incidence, timing, recurrence risk, management, triggers, including whether pregnancy or childbirth themselves may be a driver, remain uncertain

• In this woman there was a temporal association with childbirth and no alternative cause of HLH was found despite extensive screening

• We hypothesise that the return of maternal immunity following the relative immunosuppression of pregnancy may be contributory, and this hypothesis aligns with the predilection for post-partum flare seen in other inflammatory disorders including rheumatoid arthritis and systemic lupus erythematosus

• We urgently need to raise awareness and understanding of pregnancy-associated HLH:

• Following this case and others within our local MMN, we successfully sought funding for a 5-year prospective UK-wide study of pregnancy-associated HLH utilising the UK Obstetric Surveillance System (UKOSS); the study will open in 2024

